# When Visual Communication Backfires: Reactance to Three Aspects of Imagery

**DOI:** 10.1177/00936502241306707

**Published:** 2025-01-09

**Authors:** Fabienne Bünzli, James Price Dillard, Yuwei Li, Martin J. Eppler

**Affiliations:** 1 University of St.Gallen, Switzerland; 2 The Pennsylvania State University, University Park, USA

**Keywords:** imagery, reactance, environmental advocacy, persuasion

## Abstract

Although many persuasive messages include imagery, relatively little is known about the potential for the visual components to induce reactance. This research examined the effects of three message variations—camera angle (low vs. eye-level), antithesis (vs. thesis) (i.e., the juxtaposition of contrasting images), and facial expression of emotion (anger vs. happiness)—on reactance and subsequent persuasion. Two experiments (*N* = 240 and *N* = 259) using pro-environmental appeals found that variation in each of the visual features was associated with increased perception of threat to freedom, reactance and decreased persuasion. Political conservatives felt more threatened by any message than liberals, but were not differentially sensitive to image variations. This research opens the door for a programmatic analysis of imagery and reactance.

Sometimes persuasive messages not only fail to achieve their aim, they have an impact opposite of that which was intended. The motivational state that often underlies such boomerang effects is known as reactance (Brehm, 1966). To better understand this phenomenon, communication researchers have devoted considerable attention to identifying message features likely to instigate reactance (Ratcliff, 2021; Reynolds-Tylus, 2019; Rosenberg & Siegel, 2018).

Most such work has focused on *verbal* message features, such as forceful language, leaving open the question of how *visual* message features might induce reactance. This is unfortunate given that imagery has become the dominant element in modern persuasive campaigns (Kjeldsen, 2012). From these circumstances, two questions emerge: Do variations in imagery have the potential to instigate reactance? If so, what kinds of visual message features threaten people’s freedom to choose and, by doing so, arouse reactance?

This research considered three message features that might be seen as a constraint on choice: low- versus eye-level camera angle, visual antithesis versus thesis (i.e., juxtaposition of contrasting images vs. a single image), and facial expression of anger versus happiness. In what follows, we discuss the context of the investigation, review the theory of reactance, derive hypotheses, and consider a person-level moderator (i.e., political orientation), before presenting two experiments designed to test the veracity of our thinking.

## Context: Pro-environmental Appeals

Despite the millions of francs spent annually to convince Swiss residents to lower their CO2 emissions, most do not view the issue as a priority. Instead, they report a willingness to accept limits on consumption and mobility only if those restrictions are compatible with their lifestyle and do not affect their personal freedoms (SRG SSR, 2017). Although 81% indicated that they would pay higher prices for local and seasonal products, only 43% were willing to reduce their living space, and only 40% were willing to give up their car. A reluctance to make personal sacrifices may help explain why the distance travelled by cars and motorcycles in Switzerland skyrocketed by 26% since 2000, leading to a substantive increase in greenhouse gas emissions (Federal Office for the Environment FOEN, 2021). Because this is a rich, pressing, and consequential topic, our research examined messages that urged Swiss residents to reduce their carbon footprint.

## Reactance Theory

The theory of reactance consists of four causally-ordered components (Brehm, 1966). The notion of *freedom* assumes that, to varying degrees, human ability to choose is unconstrained. Anything that makes it more difficult to exercise one’s freedom to choose constitutes a *threat to freedom.* Social pressure, in the form of attempted persuasion, is the threat to freedom of greatest interest to communication science.

*Reactance* describes the “the motivational state that is hypothesized to occur when a freedom is eliminated or threatened with elimination” (Brehm & Brehm, 1981, p. 37). Reactance was initially conceptualized as a hypothetical construct that could not be measured directly (Brehm & Brehm, 1981). However, Dillard and Shen (2005) showed that reactance could be operationalized as a single construct that involves anger and critical cognitions. The underlying assumption is that people respond to freedom-threatening messages with negative feelings toward the message (i.e., anger) and cognitions critical of the message (i.e., counter-arguing or some variant thereof). Empirical findings corroborate this conceptualization (Rains, 2013).

When people feel that their freedom to choose is threatened, they become motivated to re-establish that freedom or prevent any further loss of that freedom. People may choose direct or indirect *restoration* strategies to regain a sense of autonomy. Direct restoration involves performing the forbidden behavior. Indirect restoration can take a variety of forms including derogating the source of threat (Rains, 2013), denying the threat (Worchel et al., 1976), or rejecting the message and its advocacy (Gardner & Leshner, 2016). Overall, the theory of reactance can be conceptualized as a two-step sequence (threat → reactance → [counter]persuasion) in which the effects of threat on persuasion are fully mediated by reactance (Quick & Stephenson, 2007; Rains, 2013). These two propositions have been demonstrated to hold in many investigations (Rains, 2013). We repeat them here because, together, they form a necessary condition for our subsequent predictions.

*H1: Higher levels of perceived threat to freedom correspond with higher levels of reactance (i.e., the combination of anger and critical cognitions).*
*H2: Higher levels of reactance correspond with lower levels of persuasion.*

## Prior Work on Communication and Reactance

Although many studies have examined how verbal message features bring about reactance, (Ratcliff, 2021; Reynolds-Tylus, 2019; Rosenberg & Siegel, 2018), comparatively little is known about how variations in imagery might instigate reactance. This is unfortunate in light of the widespread incorporation of images into persuasive messages (Kjeldsen, 2012) and the evidence that imagery can influence cognition and emotion (i.e., the components of reactance) (Seo & Dillard, 2019a, 2019b). One exception is the handful of anti-smoking studies that focus on the use of gruesome images to illustrate the severity of the consequences of smoking. They depict the hazards of smoking, for instance, by showing diseased lungs or an intubated patient dying of lung cancer (U.S. Food & Drug Administration (FDA), 2020). Such imagery has been linked to a variety of outcomes including threat to freedom (Hall et al., 2018), anger (Erceg-Hurn & Steed, 2011), reactance (Hall et al., 2018; LaVoie et al., 2017), and decreased persuasion (Hall et al., 2018). This set of studies make a clear and important contribution to persuasion science. Perhaps even more could be learned by examining reactance effects in a broader range of image variations.

## Imagery and Reactance

One challenge to studying imagery is classification. Images vary on a myriad of dimensions, which allows for an equal number of classification schemes (e.g., Peterson et al., 2021). To reduce this to a tractable number, we selected image variations that conformed to three criteria. (a) To ensure breadth, image features were chosen based on their conceptual distinctiveness. Building on social semiotics (Kress & van Leeuwen, 2006), we differentiated image features based on visual content information (i.e., the denotative and connotative meanings of an image), visual relational information (i.e., the relationship that is implied between the image and the audience), and compositional information (i.e., the aspects that are highlighted and made salient through visual arrangement). (b) To ensure external validity, we selected only image features that are in common use.^1^ (c) Because the smoking studies (Erceg-Hurn & Steed, 2011; Hall et al., 2018; LaVoie et al., 2017) had already explored gruesome, graphic images of individuals, we did not consider comparable photos used in environmental communication (e.g., a photo used in a campaign of the World Wildlife Fund showing a human with a fish head, suggesting that climate change will drastically change humanity). This subjective, but thorough process identified the three image features that comprise the focus of this study. We turn to them next, moving from the most subtle to the most obvious.

### Camera Angle

The first variation was camera angle, a feature that conveys information about the relationship between the image and the audience (i.e., visual relational information). Along with Kress and van Leeuwen (2006), we believe that images that contain people imply a social relationship between the model(s) and the viewer. Relatedly, many scholars have characterized human relationships in terms of two or three dimensions (Bochner & Kaminski, 1974; Dillard et al., 1999; Osgood et al., 1957). The horizontal dimension is usually interpreted as affiliation or solidarity, ranging from liking/respect to disliking/disrespect.

**Figure 1.**
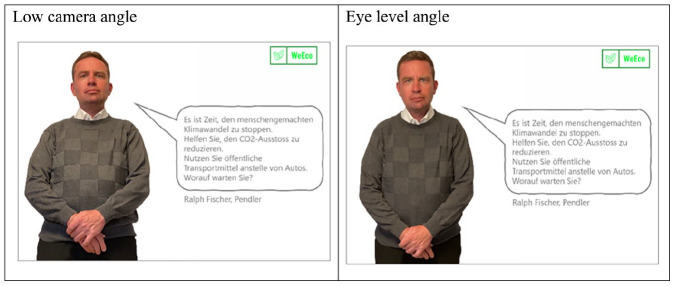
Study 1: Low angle portrait (left) vs. eye level portrait (right). *Note*. English translation of the ad copy: “It is time to stop human-made climate change. Help to reduce CO2 emissions. Use public transportation instead of cars. What are you waiting for?”

The vertical dimension represents power, where high placement on the dimension indicates dominance, low placement corresponds with submission, and eye-level placement implies equality (Kress & van Leeuwen, 2006; Macken-Horarik, 2004). This interpretation is echoed by film theorists and borne out by empirical studies showing that variation in vertical camera angle corresponds with changes in perceived dominance (Cores-Sarría et al., 2022). Specifically, speakers are rated as more potent when shown from a low camera angle (vs. eye level or high angle) (Mandell & Shaw, 1973; Sevenants & d’Ydewalle, 2006) and as more influential (Huang et al., 2002) and knowledgeable when depicted from a low angle (vs. high angle) (Tiemens, 1970). At the same time, a lower camera elicits a range of judgments that are known to cause backfire effects (Baranowski & Hecht, 2018; Hoffmann et al., 2023; McCain et al., 1977), such that speakers appear as more aggressive (Kraft, 1987) and more threatening (Fauville et al., 2022). Although these studies focus on interpersonal communication and news contexts, it is reasonable to anticipate similar effects in the realm of persuasion. When coupled with a verbal advocacy, a low camera angle—where a model looms over the viewer—signals the intent of the model to control the viewer (see Figure 1). Because any effort to constrain another can be viewed as a threat to freedom, we predicted that:

*H3a: A low camera angle (vs. eye level) portrait produces higher levels of perceived threat to freedom.*

### (Anti)thesis

**Figure 2.**
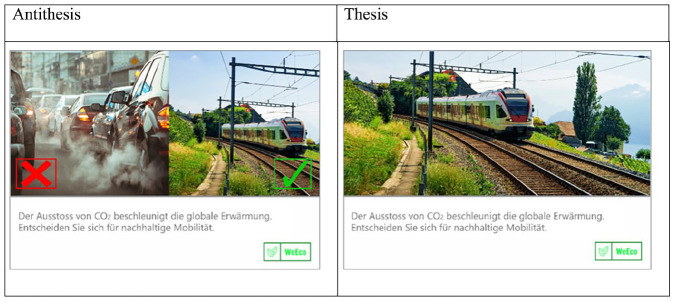
Study 1: Visual antithesis (left) vs. thesis (right). *Note*. Translation of the ad copy: “The emission of CO2 drives global warming. Choose sustainable mobility.”

A different sort of image variation involves arrangement (i.e., compositional information). How are the visual elements of an advocacy displayed relative to one another? Every persuasive appeal contains a thesis that, implicitly or explicitly, directs the viewer toward the sought-after change. Many appeals present imagery that functions as evidence. But, some messages offer a direct visual contrast between the desired and undesired outcomes. Antithesis pairs contrary images to illustrate *good versus bad* or *do versus don’t* (Huhmann, 2018; Kjeldsen, 2012; van Belle, 2009). Figure 2 provides an example.

The antithesis message in Figure 2 pairs an image of cars emitting black smoke with an image of a train driving through a verdant landscape. The X in the car image flags it as the undesirable choice, in contrast to the desirable, check-marked train. This juxtaposition of attractive versus unattractive options signals to recipients what they should or should not do. Whereas the message on the right urges reduction in CO_2_ emissions versus a universe of possible alternatives, the antithesis message reduces the decision space to a binary. From a consumer choice perspective, antitheses can be considered externally imposed limitations on people’s behaviors (Botti et al., 2008; Markman & Loewenstein, 2010; Zhang & Fitzsimons, 1999). Constraining choice has been associated with a variety of negative effects, including decreased choice-process satisfaction (Zhang & Fitzsimons, 1999) as well as non-compliance and protest behavior (Lombardini & Lankoski, 2013). Moreover, juxtaposing a desirable option with an undesirable one can be understood as a visual form of comparative advertising (Barry & Tremblay, 1975). Focusing on comparisons between brands and products, previous research has shown that comparative, versus non-comparative, advertising is perceived as more aggressive (Wilson & Muderrisoglu, 1979), more manipulative (Chang, 2007), and triggers adverse consumer responses, including counter-arguing and source derogation (Beard, 2013; Grewal et al., 1997). According to Bambauer-Sachse and Heinzle (2018a), these negative effects are the result of consumers perceiving that they are being pushed toward certain choices and, thus, can be explained by reactance theory (Bambauer-Sachse & Heinzle, 2018b, 2018a). Overall, extant literature suggests that a reduction in the proportion of options available to the decision-maker provides the grounds for reactance (Shen, 2015; Wicklund, 1974). This led us to the following hypothesis:

*H3b: A visual antithesis (vs. thesis) produces higher levels of perceived threat to freedom.*

### Models’ Emotional Expression

The third variation was facial expression of emotion, a feature that captures *what* is shown in an image (i.e., visual content information). There is a good deal of evidence that the facial expressions of models can influence the way a persuasive message is interpreted (Hatfield et al., 1994; Seo & Dillard, 2019a; Small & Verrochi, 2009). Because anger is the emotional response to the presence of an obstacle (Lazarus, 1991), an angry expression casts the viewer as a barrier to change, and the model as an agent committed to overcoming that barrier. The interpersonal relationship expressed by the message is fundamentally oppositional and choice-limiting. In contrast, a happy face signals acceptance of the viewer and a corresponding lack of intent to control. Consistent with research on nonverbal communication, we anticipated that recipients match their responses to the facial expressions displayed by a message source (Guyer et al., 2019). Accordingly, we expected people to respond to angry facial expressions of emotion with anger and counterarguing. Support for this assumption comes from research on anger appeals, suggesting that expressions of anger induce perceptions of threat to freedom as well as anger (Walter et al., 2021) and decrease overall persuasion (Kim & Cameron, 2011; Van Kleef et al., 2015; Van’t Riet et al., 2018; Walter et al., 2019). Thus, we anticipated that:

*H3c: An image that features a person with an angry facial expression (vs. happy) produces higher levels of perceived threat to freedom.*

## Political Orientation and Reactance

Audience characteristics, such as political orientation, may also influence the extent to which message recipients feel freedom-threatened by visual images. People range from conservative or right-leaning to liberal or left-leaning. Being a leading theme in contemporary conservative discourse, the idea of personal autonomy and liberty is often portrayed as a pillar of conservatism (Lupton et al., 2017). Hence, they may view *any* persuasive message as more constraining than people who identify as liberal.

Previous research lends support to this idea, especially with regard to environmental issues. One study found that Republicans (vs. Democrats) exhibited greater resistance to messages that promote support for governmental action against climate change (Zhou, 2016). Similarly, Ma et al. (2019) demonstrated that a message emphasizing scientific consensus on climate change increased reactance. But, the effect was observed only among those Republicans and Independents who believed that climate change is not actually occurring.

We considered two ways in which the coupling of ideology and perceived threat might influence the reactance process. The simple view is that conservatism unconditionally prompts higher levels of perceived threat to a persuasive message. This main effect prediction holds that, relative to liberals, conservatives over-respond to persuasive appeals and that they do so without regard to visual form.

*H4a: Political orientation is associated with higher levels of perceived threat to freedom among conservatives and lower levels among liberals.*

An alternative predicts that conservatives are uniquely sensitive to cues of freedom threat. To the extent that low camera angle, visual antithesis, and angry model expression are seen as freedom threatening conservatives will manifest greater reactance than liberals to these message variations. This expectation corresponds with a multiplicative effect.

*H4b: Political orientation interacts with freedom-threatening imagery (i.e., antithesis, low camera angle, and angry facial expression) such that conservatives feel more threatened* than liberals.

## Study 1: Method and Results

### Participants, Procedures, and Design

Swiss residents who were members of a market research panel (intervista) were invited to take part in the study if they: (a) were between 18 and 80 years of age and (b) identified as either right-leaning or left-leaning on a scale from 1 (*politically left*) to 10 (*politically right)* (i.e., people with values ranging from 1 to 3 and 8 to 10 on the political orientation scale). The initial sample included 266 participants. After screening for data quality issues (e.g., straight-lining) and non-random missing data, the final *N* was 240. Table 1 describes the sample in terms of age, gender, and education.

**Table 1.** Sociodemographic Characteristics of the Samples and the Population.

Characteristic	Study 1 sample (*N* = 240)	Study 2 sample (*N* = 259)
Age	*M* = 42.6 years	*M* = 29.6 years
Gender (female)	40.0%	48.3%
Education		
Did not complete elementary school		.8%
Compulsory school^a^	1.3%	3.5%
General or vocational education	37.9%	37.5%
Higher vocational training	27.9%	3.1 %
Higher education	32.9%	54.3 %
Other^b^		.8%

a Includes elementary school and middle school. ^b^The “other” category may encompass a variety of educational experiences, such as adult education, international qualifications recognized internationally but lacking a local equivalent, as well as informal education, including self-taught skills, online courses without formal qualifications, and informal apprenticeships.

The study tested three message features, each with a high threat and a low threat condition. The experiment can thus be understood as a 3 (message feature: camera angle vs. visual antithesis vs. facial expression of emotion) × 2 (threat: high vs. low) factorial design. Participants were exposed to both conditions of each message feature. The randomization process was structured as follows: Participants saw the three message features in random order [i.e., camera angle, (anti)thesis, facial expression]. Within each message feature, participants were randomly assigned to either the high threat condition [i.e., low shot, antithesis present, angry face] or the low threat condition [i.e., eye level shot, antithesis absent, happy face], and then the alternative condition. In this way, every participant provided data for both threat conditions for each message feature. For example, a participant who was randomly assigned to the camera angle condition was randomly assigned to the low angle condition first and then to the eye level angle condition. Once that condition was completed, the participant was randomly assigned to either the antithesis or emotion feature, then to both conditions within that message feature in random order. This process was repeated once more for the one remaining message feature. Each participant, accordingly, saw six out of six stimuli.

This relatively uncommon design has two substantial benefits, First, it generates a large number of degrees of freedom, which produces powerful statistical tests and more accurate estimates. Second, it addresses the risk of causal heterogeneity (Bullock et al., 2010), a problem to which the between-person design is blind. Although it also carries with it the risk of carryover effects, we judged the benefits as outweighing the costs. A more detailed account of our thinking is given in the Supplemental Appendix.

### Messages

The stimuli were all focused on the same topic: CO2 emissions. The verbal portion of the message (in German) asserted that cars were responsible for a large proportion of CO2 emissions, then issued a call to action to use public transportation. The text varied slightly across message features, but was identical within each message feature for the high and low threat condition (for the German translation of the messages, see the notes in Figures 1–2). For the camera-angle stimuli (Figure 1), we did a photo shoot with a friend of one of the authors. Two photos were taken simultaneously—one from below and the other at eye level—to ensure that the images differed only in terms of angle. The photos for the antithesis conditions were drawn from a commercial image database (Figure 2). For the emotionally-expressive imagery, we used an pre-validated set of images showing the same man once with an angry facial expression and once with a happy facial expression (Ebner et al., 2010).

A qualitative pre-test (*N* = 10) was conducted to assess the visuals. The participants were shown each pair (e.g., happy vs. angry) and asked about their perceptions (we especially focused on the extent to which they perceive the visuals as controlling). Responses indicated that the visuals were perceived to differ in the extent to which they threaten freedom to choose.

### Procedure

The experiment was conducted in June 2021. Following consent, participants were directed to a survey that included questions about sociodemographics (e.g., age, sex, education, political orientation), followed by the three experimental conditions, all of which included questions about their reactions to each message (e.g., perceived threat to freedom).

### Measures

#### Political Orientation

This item utilized a 10-point scale anchored at 1 (=*politically left*) and 10 (=*politically right*). Following the screening process described above, respondents were classified as either left (i.e., scores of 1–3) or right (i.e., scores of 8–10). This liberal/conservative binary variable was used in subsequent analyses.

#### Perceived Threat to Freedom

Perceived threat to freedom was measured with the average of three items whose response scale ranged from 1 (*strongly disagree*) to 5 (*strongly agree*) (Dillard & Shen, 2005): *The campaign image tried to make a decision for me, The campaign image tried to manipulate me*, and *The campaign image tried to pressure me*, (*M* = 2.341, *SD* = 1.317, α = .924). We used a subset of the four items in Dillard and Shen (2005) because the factor loadings and fit statistics in our previous studies showed that a valid and reliable estimate could be obtained with just three items.^2^ The smaller set was intended to minimize respondent fatigue.

#### Reactance

This variable was indexed as a combination of anger and critical cognitions. Participants were asked to report how *angry, annoyed*, and *aggravated* they felt when viewing the campaign image (Dillard & Shen, 2005). The five-point response scale was anchored at 1 = *none of this feeling* and 5 = *a great deal of this feeling*, (*M* = 1.924, *SD* = 1.188, α = .961). To assess critical cognition, participants were asked to type in whatever thoughts they had when viewing the campaign images. The resulting data were coded in a four-step process by the first and fourth authors. For each step, they classified a subset of the data (15% of the sample), then estimated the reliability of the coding procedure at that step. Disagreements were discussed and resolved before the first author classified the remaining data.

In step one, the coders segmented the responses into thought units (i.e., independent clauses; (Hatfield & Weider-Hatfield, 1978). Agreement on segmentation was 95.6%. Second, cognitive responses were separated from affective responses. Data containing affective expressions were filtered out (e.g., sad, frustrated, happy, amusing, annoying, embarrassing). Using Krippendorff’s alpha, reliability was .85 (Hayes & Krippendorff, 2007). The third step involved judging the remaining thoughts as relevant or irrelevant. Relevant thoughts were defined as responses that were related to the message topic (e.g., “climate change is real”), whereas irrelevant thoughts were defined as those that had no logical relationship to the message topic (e.g., “Politicians do whatever they want”). α_K_ was .85 for this step. Ultimately, relevant thoughts were classified as either supportive (e.g., “More people should use public transportation to reduce CO2 emissions”), neutral (e.g., “this campaign is about an environmental issue“), or critical (e.g., “I do not let anyone tell me how to travel”). α_K_ was 0.86. Only the negative thoughts were included in the subsequent analyses. On average, participants generated .590 negative thoughts after viewing one message (*SD* = .834).

Given strong evidence that anger and critical cognitions form an amalgam that is indicative of reactance (Rains, 2013), they were combined into a single variable. To ensure that they were equally weighted, z scores were computed for each, then averaged (*M* = 0.000, *SD* = 1.565).

#### Perceived effectiveness (PE)

Three 7-point semantic differentials measured perceived message effectiveness: *not at all persuasive–very persuasive, not at all convincing–very convincing, not at all effective–very effective* (Seo et al., 2013), *M* = 3.558, *SD* = 1.982, α = .940). This index was preferred over more traditional measures of persuasion because our earlier research on environmental topics produced distributions that were highly skewed, thereby making range restriction a concern (Bünzli & Dillard, 2022).

### Plan for Analysis

The reactance process was examined via structural equation modelling in Mplus 8.10 (Muthén & Muthén, 1998–2017). Because preliminary analyses revealed substantially non-normal distributions (Supplemental Figure S1), we utilized the maximum likelihood routine with robust standard errors (MLR). Initially, we treated all endogenous variables as latent and individually corrected each one for measurement error. When this approach produced non-positive definite results, the model was simplified such that the endogenous variables were made manifest. This produced a proper solution and interpretable results.

**Figure 3.**
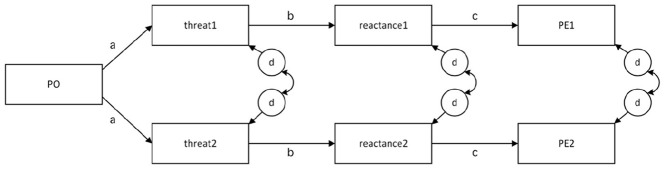
Study 1: Abbreviated path diagram. *Note*. For simplicity, only one message condition is shown here (e.g., antithesis vs. thesis). The actual model included threat, reactance, and perceived effectiveness across all six cells. PO = Political Orientation. PE = Perceived Effectiveness. The letters a, b, and c indicate paths that were initially constrained to equality (see Table 1). The circled ds are disturbance terms, which were allowed to covary because the design is repeated measures. Each d term was freely estimated.

The base model was represented by the predictions of H1 (threat causes reactance) and H2 (reactance causes perceived effectiveness) (Figure 3). Because participants provided data for each cell in the design, we created this X → Y → Z model six times, once for each cell. To evaluate the degree to which the reactance process was homogeneous across conditions, all threat-to-reactance paths were set to equality and all reactance-to-PE paths were set to equality. To test H3a-3c, which focused on differences in threat within image type conditions, we estimated cell means and tested for differences (e.g., low angle vs. eye level).

Political orientation was added as a predictor of threat1 to threat 6. Because political orientation was measured once at the person level, it was positioned as the exogenous variable causing the message-level processes described above. Consistent with H4a, the initial model constrained all paths from this variable to be equal. Another model was tested in which all three paths to the less-threatening message features (i.e., eye level, thesis, happy) were constrained to equality and all three paths to the high-threatening features (low angle, antithesis, angry) were constrained to equality. This tested the interaction predicted by H4b.

The disturbance terms for endogenous variables were allowed to correlate with the disturbance terms of other endogenous variables of their same type. Put differently, all possible correlations were allowed among disturbance terms for reactance1 to reactance6 and among disturbance terms for PE1 to PE6.

Model fit was evaluated using the following criteria: CFI > .90, RMSEA < .08, SRMR < .10 (Hu & Bentler, 1998; Kline, 2016). Scaled χ^2^ difference test (Satorra, 2000) and BIC change were used to compare models. We followed Raftery’s (Raftery, 1995) criteria for interpreting BIC differences: Negative values are preferred and differences of 0–2 are considered weak evidence of superiority, 2–6 as positive evidence, 6–10 as strong evidence, and values >10 as very strong evidence.

### Model Fitting

Before meaningful tests of the hypotheses can be considered, it is necessary to identify models that adequately reproduce the structure of the data. Accordingly, we began the process of model fitting with a restrictive model, then systematically freed paths until we achieved a balance between theory and fit. Model 1 constrained all instances of the same conceptual path to be equal (Figure 3). That is, one coefficient was estimated for political orientation → threat, one for threat → reactance, and one for reactance → PE across all six messages. As the first row of Table 2 shows, this fully restrictive model showed poor fit. Next, we considered whether the reactance process differed by image type. We constructed three models, each of which constrained threat → reactance to equality and reactance → PE to equality within one image type condition while holding the corresponding paths equal in the other two conditions (e.g., low camera angle vs. eye level). As shown in Table 2, Model 4 was not only superior to Models 2 and 3 on all fit indices, it represented a huge improvement over Model 1 (Satorra-BentlerΔ*χ*^2^(2) = 30.289, *p* < .001; ΔBIC = −−33.372). By contrast, the release of equality constraints in Models 2 and 3 did not substantially improve model fit. Each produced a nonsignificant reduction in *χ*^2^ and an increase in BIC. These results suggested that the reactance process was equipotent in the camera angle and facial expression conditions, but different in the (anti)thesis conditions.

**Table 2.** Study 1: Fit Statistics for Path Models.

Model		χ2	DF	S-BΔχ2	CFI	TLI	RMSEA	90CI	pCLOSE	SRMR	BIC	ΔBIC
1	Fully restrictive model	260.388***	123	—	.937	.913	.068	[.057, .080]	.006	.122	13572.489	—
2	Non-equivalent: Camera angle	251.837***	121	8.158*	.940	.916	.067	[.055, .079]	.009	.121	13574.087	1.598
3	Non-equivalent: Expression	257.545***	121	3.081	.938	.912	.069	[.057, .080]	.005	.121	13579.580	7.091
4	Non-equivalent: Antithesis	219.460***	121	30.289***	.955	.936	.058	[.046, .070]	.133	.114	13539.117	−33.372
4a	Non-equivalent: Antithesis Reactance → PE distinct	220.163***	122	25.047***	.955	.937	.058	[.045, .070]	.141	.115	13534.659	−37.830
4b	Non-equivalent: Antithesis Threat → reactance distinct	259.727***	122	.854	.937	.912	.069	[.057, .080]	.005	.121	13576.946	4.457
5	Non-equivalent: All features distinct	219.596***	119	1.062	.954	.934	.059	[.047, .072]	.104	.114	13549.876	15.217
6	Non-equivalent: All images distinct	196.261***	108	23.914*	.960	.936	.058	[.045, .071]	.142	.109	13586.297	51.638
7	Non-equivalent: Antithesis Reactance → PE distinct PO → threat contrast	219.906***	121	.028	.955	.936	.058	[.046, .071]	.129	.115	13540.125	5.466

*Note.* Fit statistics of the underlined models were used for contrasts in the following block divided by a black line (i.e., χ2 and BIC differences for models 2–4 were contrasted against model 1; models 5–7 were contrasted against model 4a). Model 7 replaced equality constraints on political orientation→threat for all message conditions with two constraints on the same path, for image conditions that were more threat-inducing versus images that were less threat-inducing, respectively.

* *p* < .05. ***p < .001.

Next, we sought to identify the theoretical path that drove the improvement in fit. We conducted two Wald tests based on Model 4 to evaluate the two equality constraints on antithesis/thesis conditions in Model 4. Specifically, we constrained the threat → reactance path to be equal across all conditions, but allowed the reactance → PE path in the antithesis condition to be different from other image types in Model 4a. Conversely, in Model 4b, reactance → PE was constrained to be equal across conditions, and two estimates were given for the threat → reactance path in antithesis versus remaining image types. Wald tests suggested Models 4 and 4a were indistinguishable (Wald *χ*^2^(1) = .865, *p* = .355), while the additional constraint on Model 4b increased model misfit (Wald *χ*^2^(1) = 35.026, *p* < .001). These results led us to interpret coefficient estimates in Model 4a. It indicated that most theoretical paths were equivalent across image type conditions, but that reactance → PE was different in the antithesis/thesis conditions.

The next set of model comparisons further affirmed non-equivalence among conditions. In particular, treating the reactance process as distinct across three image types (Model 5) or across six images (Model 6), or freeing the equality constraints on threat → reactance and reactance → PE paths on the basis of Model 4a, did not result in substantive improvements in fit. Modifying Model 4a into Model 5 resulted in a miniscule reduction of *χ*^2^ (Satorra-BentlerΔ*χ*^2^(3) = 1.062, *p* = .786), and an increase in BIC (ΔBIC = 15.217). While Model 6 resulted in a significant reduction in *χ*^2^ (Satorra-BentlerΔ*χ*^2^(14) = 23.914, *p* < .05), BIC change (ΔBIC = 51.638) suggested the model was too complex (i.e., too many free parameters).

Finally, to test H4b, we considered whether the extent to which political orientation’s influence on perceived threat to freedom was different across all images. We constructed Model 7 by modifying the equality constraint on PO → threat in Model 4a into constraints that set this path to be equal between messages that included more threatening visual cues (low angle, antithesis, angry facial expression) and between those that did not (eye level, thesis, happy facial expression), respectively. This modification resulted in a nonsignificant reduction in the Satorra-Bentler *χ*^2^(1) = .028, *p* = .867 and a nontrivial increase in BIC of 5.466 (Raftery, 1995). Thus, there was no indication that relaxing these constraints improved model fit.

The series of model contrasts led us to tentatively accept Model 4a. The distinctive features of this model were that it constrained (a) PO → threat and threat → reactance to be equal across all conditions, and (b) reactance → PE to be equal in the camera angle and emotional expression conditions, but distinct from the antithesis type condition.

Although Model 4a demonstrated good fit to the data, we also tested two alternative models, each of which varied the ordering of variables in the two step reactance process (see Supplemental Appendix for results). The data showed that the causal ordering of variables in Model 4a was superior to both alternative models.

### Hypothesis Testing

#### H1-2: The Core Reactance Process

Hypotheses 1 and 2 predicted that higher levels of perceived threat to freedom would correspond with greater reactance, which would hinder persuasion. Both hypotheses were supported. For H1, threat was a significant and positive predictor of reactance in all conditions (all *p* < .001). As shown in the top panel of Table 3, the standardized coefficients ranged from β = .375 to .486. The unstandardized coefficient and its 95% confidence interval also appear in the same panel. For H2, reactance significantly and negatively predicted perceived effectiveness (all β = −.521~−.211, all *p* < .001) in all conditions. However, the reactance → PE path was significantly stronger in the antithesis/thesis conditions than in the camera angle or facial expression conditions (Table 3).

**Table 3.** Study 1: Path Coefficients and Variability Indices.

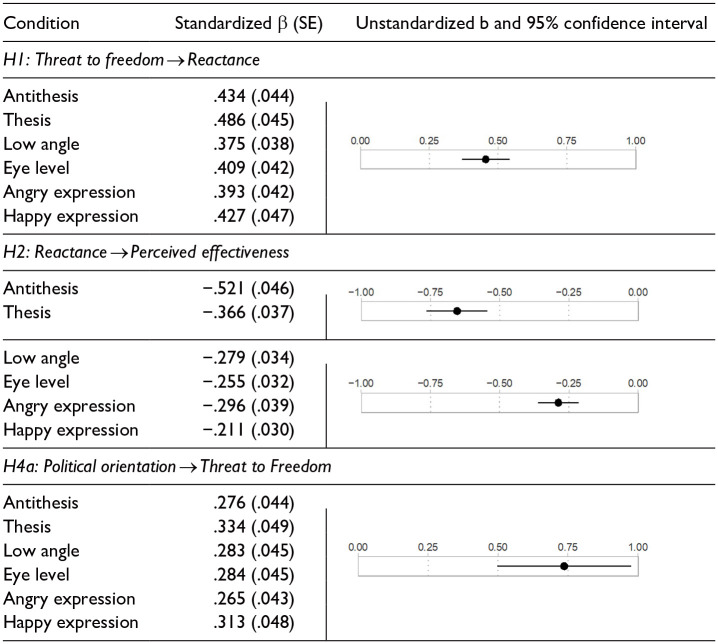

*Note.* Conditions that shared the same unstandardized coefficient estimates (and corresponding C.I.s) for a given path were under equality constraint for that path. Standardized coefficients differed due to different variances in measured variables. Political orientation was scaled as conservatives = 1, liberals = 0. All paths *p* < .001.

#### H3: Visual Instigators of the Reactance Process

Mean comparisons were estimated in Model 4a, which should be interpreted on the 5-point scale used to measure perceived threat (see Table 4).^3^ Compared to the eye-level image, the low-angle image elicited greater perceived threat to freedom (∆_*Mcamera angle*_ = .250, *p* < .001, 95%CI = [.143, .357]). This result was consistent with H3a. In line with H3b, visual antithesis was more threat-inducing than visual thesis (∆_*Mantithesis*_ = .771, *p* < .001, 95%CI = [.628, .913]). Similarly, the image of an angry model was more threatening than the image that featured the same model with a happy expression (∆_*Mexpression*_ = .553, *p* < .001, 95%CI = [.409, .697]). This confirmed H3c. Additional tests of the impact of visual variations on perceived threat using a between-persons approach are given in the Supplemental Appendix.

**Table 4.** Study 1: Means and Mean Differences in Threat Between Image Conditions.

Image condition	*M* (*SD*)	*ΔM*
Low angle	2.612 (1.317)	.250
Eye-level	2.362 (1.309)	
Antithesis	2.576 (1.340)	.771
Thesis	1.806 (1.078)	
Angry expression	2.622 (1.413)	.553
Happy expression	2.069 (1.210)	

*Note*. Threat was measured on a 1-5 scale where higher values indicate higher levels of perceived threat to freedom. All mean differences *p* < .001.

#### H4: Political Orientation

H4a predicted that the extent to which one feels threatened by a pro-environmental message would vary as a function of political orientation. Across conditions, conservatives perceived greater threat than liberals (unstandardized b = .736, 95%CI = [.498, .975]). The standardized path coefficients within each image condition also showed significant and positive effects of conservatism on threat (βs = .265~.334, all *p* < .001, Table 3).

Hypothesis 4b predicted that imagery would interact with political orientation to predict perceived threat to freedom. For this, we contrasted Model 7 against Model 4a (discussed above) and found that allowing the slope of PO → threat to differ between more and less threat-inducing images did not improve model fit. Hence, there was no evidence of an interaction between political orientation and image type on threat. H4b was not supported.

## Study 2: Method and Results

To corroborate the findings from the initial study, we conducted a replication. The study again focused on environmental advocacy but added a between-subjects element to the randomization process to counter potential demand effects. Moreover, internal replications were used for each message feature to further strengthen the generalizability of the findings.

### Participants, Procedures, and Design

Members of a market research panel (Prolific) were invited to participate in the study if they: (a) lived in Switzerland, Germany, Austria, or Lichtenstein, (b) were fluent in German, and (c) were at least 18 years old. The initial sample included 261 participants. After screening for data quality and non-random missing data, the final *N* was 259. Table 1 describes the sample in terms of age, gender, and education.

The experiment tested three message features, each with a high and a low threat condition, which in turn consisted of two stimuli each. The experiment thus can be described as a 3 (Message feature: camera angle vs. visual antithesis vs. facial expression of emotion) X 2 (Threat: high vs. low) X 2 (Version: stimulus 1 vs. stimulus 2) factorial design. Whereas message feature and version were within-subjects factors, threat was a between-subjects factor. Participants were exposed to all three message features in random order [i.e., camera angle, (anti)thesis, emotion]. Within each message feature, they were randomly assigned to *either* the high threat condition [i.e., low shot, antithesis present, angry face] or the low threat condition [i.e., eye level shot, antithesis absent, happy face]. Subsequently, participants were presented with two stimuli within the assigned condition, displayed in random order. For instance, a participant who was randomly assigned to the antithesis condition was randomly placed in the high-threat condition, where she saw two antithesis ads in random order. After completing this condition, the participant was randomly assigned to the emotion variable, followed by a random assignment to the low-threat condition. Subsequently, she saw the two angry face ads, presented in random order. This process repeated once again for the remaining message variable (i.e., camera angle).

Each participant, accordingly, saw six out of twelve stimuli. We assigned participants to either a high or low threat condition for each feature to counter the potential for demand effects. Because no one saw both threat conditions within the same message feature, the effects are between-subjects.

### Messages

The stimuli focused on mobility and climate change. The verbal portion of the message (in German) emphasized the negative impact of CO2 emissions from fossil-fueled transportation on climate change and urged individuals to use sustainable modes of transportation. The text varied slightly across message features but was identical within each message feature for the high and low threat conditions (for the German translation of the messages, see the notes in Supplemental Figures S2–S4). For the camera-angle stimuli (Supplemental Figure S2), we did a photo shoot with one of the authors and one colleague. Two photos were taken simultaneously—one from below and the other at eye level—to ensure that the images differed only in terms of angle. The photos for the antitheses conditions were drawn from commercial image databases (Supplemental Figure S3). For the emotionally-expressive images (Supplemental Figure S4), we used an pre-validated set of images showing a man and a woman once with an angry facial expression and once with a happy facial expression (Conley et al., 2018).

### Procedure

The experiment was conducted in March 2024. Following consent, participants were directed to a survey that included questions about sociodemographics. Subsequently, they were exposed to six stimuli, after each they were asked about their reactions to that message (e.g., perceived threat to freedom, anger, counter-arguing, persuasion).

### Measures

*Political orientation* was assessed on a 5-point scale, ranging from 1 = *Very Liberal*, 2 = *Liberal*, 3 = *Middle of the road*, 4 = *Conservative* to, 5 = *Very Conservative* (*M* = 2.444, *SD* = .843). We assessed *perceived threat to freedom* (*M* = 2.537, *SD* = 1.188, ω_within_ = .872, ω_between_ = .943), *anger* (*M* = 2.065, *SD* = 1.094), and *perceived effectiveness* (*M* = 3.690, *SD* = 1.652, ω_within_ = .928, ω_between_ = .869). The thought-listing measure for *critical cognition* was replaced with a two-item scale-based measure. Where 1 = *Not at all* to 5 = *Very much*, participants were asked how *critical* and *negative* their thoughts were when viewing the campaign message (*M* = 2.639, *SD* = 1.150). *Reactance* was then indexed as a combination of anger and critical cognitions (ω_within_ = .898, ω_between_ = .948).

### Plan for Analysis

Because each participant generated responses for six out of twelve stimuli, responses are nested within individuals. As such, we used a two-level structural equation model to test our hypotheses: level 1 focused on message-level, within-individual processes, and level 2 examining between-subject processes. Before fitting such a model, we conducted measurement analysis on the multi-item scales used in the study. The multilevel confirmatory factor model was a good fit to the data. χ^2^(120) = 537.158, *p* < .001, RMSEA = .047, CFI = .953, SRMR_within_ = .045, SRMR_between_ = .079.

We proceeded to hypothesis testing by specifying structural paths among latent variables and including exogenous variables at both levels. The core reactance process functions at the message level and the person level. At the message level, variations in images (i.e., threat × image type) are examined as instigators of the reactance process, and we included a viewing sequence variable to examine and control for order effects on the reactance process (first image = 1, last image = 6). At the person level, we investigate the extent to which political orientation influences the core reactance process. All models were estimated with the MLR estimator in Mplus 8.10 (Muthén & Muthén, 1998-2017).

### Model Fitting

Because there were six levels of experimental manipulation, multiple variables are needed to represent image variations at the message level. Specifically, we included dummy-coded variables for high- versus low-threat conditions across image variations and two of the image types (i.e., antithesis and emotion), as well as their interaction terms (antithesis variation × threat condition identifies the presence of the antithesis composition; emotion variation × threat condition identifies the use of anger image; see Supplemental Table S4 for stimuli coding). These five binary variables were necessary to represent all levels in our 3 × 2 design. Configured this way, the eye-level images served as the referent level in the model. In an initial model, we specified that image variations and viewing order predicted threat, threat predicted reactance, and reactance predicted perceived effectiveness at the message level; at the person level, political orientation predicted the core reactance process. This model was not a great fit to the data, χ^2^(208) = 1149.310, *p* < .001, RMSEA = .054, CFI = .911, SRMR_within_ = .078, SRMR_between_ = .080, BIC = 48983.281. Modification indices suggested image variables exerted a pervasive influence on the entire core reactance process beyond threat; we added paths from variables representing image variations and viewing order to reactance and perceived effectiveness at the message level. This modified model fit the data well, χ^2^(196) = 795.084, *p* < .001, RMSEA = .044, CFI = .943, SRMR_within_ = .042, SRMR_between_ = .071, BIC = 48560.886; Δχ^2^(12) = 323.547, *p* < .001, ΔBIC = -422.395. We obtained standardized coefficients from this model to test H1–H4a. For H4b, we constructed a separate, two-level random effects model (BIC = 49131.701), specifying the path from image threat condition (high vs. low threat) to perceived threat at the message level as a random slope, and we used the political orientation variable at the person level to predict this random slope. Because parameter standardization and fit statistics are not available for a random effects model, we used the significance test associated with the unstandardized coefficient to test H4.

### Hypothesis Testing

#### H1-2: The Core Reactance Process

H1 and H2 evaluated the tenability of the reactance process (i.e., threat → reactance → counter-persuasion). This process was tested at both message and person levels of the model. At the message level, threat was a positive predictor of reactance (standardized β = .528, *p* < .001), which in turn hampered persuasion (β = −.587, *p* < .001). At the person level, the same pattern holds: threat increased reactance (β = .806, *p* < .001), and reactance decreased perceived message effectiveness (β = −.492, *p* < .001). H1 and H2 were both supported.

#### H3: Visual Instigators of the Reactance Process

As noted previously, we investigated the effects of visual features on the reactance process, controlling for the order in which stimuli were viewed. Image position exerted a direct effect on threat, reactance, and perceived effectiveness; in general, images viewed later in the experiment were rated as less threatening (β = −.109, *p* < .001), but induced more reactance (β = .073, *p* = .006), and they were viewed as more effective (β = .180, *p* < .001).

Controlling for order, variables representing the experimental conditions collectively predicted threat, reactance, and perceived effectiveness. The standardized coefficients for these parameters are summarized in Table 5. Because our interest was in testing the extent to which image variations cause differences in perceived threat to freedom, we used the unstandardized parameter estimates to compute contrasts in measured threat and test H3a–H3c.

**Table 5.** Study 2: Standardized Coefficients for DV.

IV	Standardized coefficient (β) for DV		
Message level	Threat	Reactance	Perceived effectiveness
High Threat Image	.389***	.201**	.106*
Antithesis Variation	−.398***	−.332***	.369***
Emotion Variation	−.219***	−.175**	.181***
Antithesis Present	−.013 ns	−.080 ns	−.087 ns
Emotion Anger	.116†	.084 ns	−.135*
Viewing Order (1 = first, 6 = last)	−.109***	.073**	.108***
Threat		.528***	
Reactance			−.587***
Person level	Threat	Reactance	Perceived effectiveness
Conservatism	.388***		
Threat		.806***	
Reactance			−.492***

*Note*. The eye-level images serve as the referent level (see Table 5 for stimuli coding). Threat and Reactance listed in rows reflect their function as predictors, and listed in columns, as dependent variables.

† *p* < .10. *p < .05. **p < .01. ***p < .001.

These contrasts show the high threat condition for each image variation induced greater perceived threat to freedom than its low threat counterpart. The low-angle shot yielded greater threat to freedom than the eye-level shot (ΔΜ = .474, *p* < .001), supporting H3a. Similarly, antithesis was more threatening than thesis (ΔΜ = .454, *p* < .001), supporting H3b. H3c was confirmed: the angry model elicited greater freedom threat than the happy model (ΔΜ = .663, *p* < .001).

#### H4: Political Orientation

H4a predicted political conservatives would feel more threatened by pro-environmental messages than liberals. Results confirmed this prediction: conservatism was positively associated with perceived threat to freedom (β = .388, *p* < .001). In a separate, random effects model, political orientation did not predict the variability in this path (unstandardized b = .047, *p* = .432). H4b was not supported.

## Discussion

The results of two experiments showed that all three image variations influenced perceived threat, reactance, and persuasion in ways predicted by the theory. Although conservatives felt more threatened by the messages than did liberals, there was no indication that they were especially sensitive to the visual variations of interest.

### Visual Instigators of the Reactance Process

#### Camera Angle

We examined three different image variations, one of which altered the model-viewer relationship by changing the camera angle. This was appealing for its operational simplicity and because it so readily mapped onto structural models of interpersonal relationships. The within-person findings of both experiments were compatible with the characterization of low camera angle as “a subtle threat cue” (Elliot et al., 2011, p. 1340) and with the notion that vertical angle can be understood as a dominance bid.

Nevertheless, it is obvious that our work barely scratched the range of theoretical possibilities implied by dimensional conceptualizations of interpersonal relationships. A straightforward extension of the current studies might employ images that vary along the full range of the vertical-power dimension. This would include a high camera angle (from the viewer’s perspective), that signals a position of viewer power and implies a correspondingly low level of perceived threat to freedom. Further, the dimensional models identify a horizontal solidarity/affiliation axis that deserves attention too (Bochner & Kaminski, 1974; Osgood et al., 1957). The theoretical mapping of camera angle onto the horizontal (vs. vertical) dimension is less intuitive. Creating testable predictions may require factoring in context or the emotional expression of the model. Finally, model-viewer proximity might be conceptualized as the often-recovered third dimension of relationships, which travels under labels such as immediacy and involvement (Andersen et al., 1998; Cegala et al., 1982). If proximity functions to amplify relational judgments, as suggested by one theory (Dillard et al., 1999), we should expect it to interact with the location of the model on the vertical and/or horizontal dimensions.

#### (Anti)thesis

Our second image variation focused on the juxtaposition of contrasting images. We expected that anti-thesis (vs. thesis) would be seen as choice restrictive because it limits the decision space to a binary, instantiated as two opposing images: One versus the other. Antithesis was compared to the single image condition (i.e., thesis), which implies some ill-defined, but open-ended decision space: One image versus whatever else comes to mind, including nothing. Findings from both experiments support this prediction, which aligns roughly with the position that reactance is a function of the proportion of freedoms that are threatened (Wicklund, 1974, chapter 6). We say “roughly” because the mathematics of proportions are not suited to open-ended decision spaces; Proportions are point estimates, not ranges. Knowledge of how message recipients subjectively conceive of the options implied by a thesis image would speak to the how-to-compute-a-proportion problem. The issue can be expressed in this way: As researchers, we want to compute a proportion so that we can test Wicklund’s prediction. To do this we need specific values for the numerator and denominator, something that is made difficult by the open-ended nature of thesis images. A message that says only “Choose door #1” (the numerator) does not reveal how many other doors might exist. Does the viewer conceive of the decision as choose door #1 versus not? Or as door #1 versus some large, but unspecified number of alternative doors? Only by gaining insight into how viewers fill in the missing denominator can we test the prediction that reactance is determined by the proportion of freedoms that are threatened.

It is also worth mentioning that antitheses can be accomplished in many ways – contrasting colors, forms, shapes, angles, lights, as well as visual content (Davison, 2002). To simplify inquiry, we offer a person-focused mini-theory of antithesis, premised on the fact that anti thesis messages have two parts. The *options component* illustrates and contrasts possible states. In Figure 2, the traffic image and the train image are the options. They vary individually in terms of *plausibility*, that is, the degree of perceived correspondence with the real world. Is the image a believable representation or is it biased, extreme, or unrepresentative?

The images also vary jointly in terms of *discrepancy* or the subjective distance between the two. An antithesis might display alternatives that are similar to one another or starkly different. We expect that plausibility is inversely related to perceived threat to freedom, such that the more biased, extreme, or unrepresentative an option, the greater the perceived threat to freedom. In contrast, we anticipate that discrepancy is directly associated with threat, such that the greater the contrast between the alternatives, the more people feel threatened in their autonomy.

The *directive component(s)* articulate(s) the preferred and/or nonpreferred choice. Figure 2 contains two directives, one that says Yes (i.e., the checkmark) and one that says No (i.e., the X). Logically, either one alone would be sufficient to make the same point and might induce less perceived threat. Or the use of a question might steer message recipients toward the preferred option more subtly and with reduced threat (e.g., “Which would you choose?”). Further, we might expect that perceived threat would be heightened by more directives, exclamatory markers (e.g., larger fonts, capital letters, or exclamation marks), and certain colors (Armstrong et al., 2021, for evidence of color effects). Though our thinking here goes well beyond the data, we hope that these concepts provide traction for future inquiry.

#### Models’ Emotional Expression

The third image variation turned attention to the photo models. In this case, we considered emotional expression, a content property of the image that communicated an agent-versus-obstacle relationship in the anger condition and an agent-versus-acceptance position in the happy condition. Results were consistent with expectations: The anger expression elicited higher levels of perceived threat than the happy expression.

There are many forms of emotional expression not included in our research and a correspondingly large number of avenues for future research. For example, the view that emotional expressions are culturally invariant holds that there are six basic facial expressions: Surprise, fear, disgust, anger, happiness, and sadness (Ekman & Friesen, 1971). While we take no position here on the number or the invariance of these expressions, future research should consider comparisons beyond anger and happiness. The basic emotions position is complemented (some would say opposed) by evidence that comprehension of emotional expression depends on various contextual cues: (a) the pre-message affective state of the viewer, (b) culturally-meaningful background imagery, as well as (c) verbal labeling of the expression itself (Barrett et al., 2011). It should be noted that these are *categories* of context cues, each of which contain myriad variations. Together the two positions point toward a rich array of opportunities for researching expressions and reactance.

### Political Orientation

Around the globe, contemporary conservative political discourse emphasizes the notion of personal freedom — an observation that suggests that conservatives may be more inclined than liberals to see any persuasive message as autonomy-threatening. And while our messages were focused only on climate change, we did find a propensity for right-leaning people to feel more threatened than left-leaning persons. This is consistent with other research showing conservatives to be more resistant to persuasion generally (Ma et al., 2019; J. Zhou, 2016) and with respect to the pro-environmental advocacies specifically (Cousse et al., 2020).

We also examined the possibility that conservatives might be especially sensitive to freedom-threatening visuals, but our data gave no indication that this was the case. To the extent that this result generalizes beyond our study, it is good news for pro-environmental message designers. Although conservatives may still tilt away from pro-environmental action, any concern that visual variations will alienate them disproportionately may be misplaced. If true, limited resources for climate-related persuasion need not be used to develop specialized campaigns that are adapted to uniquely reactance-prone audiences. However, given the power of political identity in shaping responses to climate change, additional research is needed.

### The Core Reactance Process

This project is one of many that confirm the two-step sequence in which perceived threat causes increases in reactance and that reactance brings about diminished persuasion (Quick & Stephenson, 2007; Rains, 2013). Our results go beyond pure replication by making two methodological contributions to that body of work. One is the explicit testing and elimination of alternative causal orderings of threat, reactance, and persuasion (Supplemental Appendix: Study 1). We are not the first researchers to do this (cf., Rains & Mitchell Turner, 2007), but strong evidence of mediation requires multiple investigations. Repeated rejection of rival models is one way in which the case for statistical mediation is advanced (e.g., Iacobucci et al., 2007). We suggest making evaluation of alternative models a standard practice (see Zhou et al. (2024) for a more extensive treatment of this issue).

Framing the second contribution begins with the premise that confidence in scientific conclusions is increased when the same results are obtained across a variety of conditions. If an effect is observed in multiple laboratories (vs. one) or by multiple studies (vs. one), this diversity strengthens our confidence in the finding. Of course, this principle applies to other research designs as well. To the best of our knowledge, our experiments are among the first to examine the reactance process using something other than between-persons design (for another see Gardner & Leshner (2016)). To clarify the value added by our project, it is helpful to compare the effect sizes with those reported in Rains’ (2013) meta-analysis: .37 for threat→reactance (vs. .37 to .48 in Study 1) and −.30 for reactance → persuasion^4^ (vs. −.21 to −.52 in Study 1). Even though the two sets of results derive from different research designs, which embody different configurations of threats to internal validity, they are quite similar. When comparable findings derive from diverse designs, we should have increased faith that they reflect a durable feature of human behavior. More detail on the risks and benefits of our design is given in the Supplemental Appendix.

### Strengths and Limitations

The most notable limitation of this report concerns our focus on a single advocacy domain, that is, environmental advocacy. Further research is needed to verify if the results generalize to other domains such as health or social justice.

Second, while our two experimental studies demonstrate that images have the potential to evoke reactance, we should note that this assertion does not hold for every member of our sample. For instance, roughly 35% (500/1,440) of our sample in Study 1 indicated that they experienced a value of 0 for threat and for reactance. In other words, approximately 65% *did* experience reactance and 35% *did not* (Supplemental Figure S1). To some readers, the latter value may appear large. In our view, it is remarkable that single-frame, still images were able to induce reactance in 2/3 s of participants.^5^

Third, in both studies we utilized a design that put every participant in several cells. This greatly increased our ability to detect differences and it addressed the problem of causal heterogeneity (Bullock et al., 2010). The price of these benefits was the possibility of carryover effects, and there was some evidence of small effects in both experiments (the Supplemental Appendix provides a detailed treatment of this issue). However, controlling for such effects in Study 2 did not change either the significance or the direction of the visual message effects under study. Moreover, our designs randomized participants to all possible orders. To the extent that carryover effects were present, they should have been equally distributed across conditions and, therefore, not a systematic source of bias. And because all participants were evenly allocated to all orders, there was no possibility that carryover effects associated with any particular order was unequally weighted relative to any other order.

## Conclusion

Why do people resist persuasion? Based on two experiments, we now know that certain image types have the potential to evoke reactance. Camera angle, arrangement, and model emotional expression are all implicated in the reactance process. The results have immediate applied implications for the design of environmental appeals. We hope that they also stimulate inquiry into the profoundly interesting questions that lie at the intersection of imagery and persuasion.

## Supplemental Material

sj-docx-1-crx-10.1177_00936502241306707 – Supplemental material for When Visual Communication Backfires: Reactance to Three Aspects of Imagery Supplemental material, sj-docx-1-crx-10.1177_00936502241306707 for When Visual Communication Backfires: Reactance to Three Aspects of Imagery by Fabienne Bünzli, James Price Dillard, Yuwei Li and Martin J. Eppler in Communication Research

## Notes

1. The first author, who possesses content expertise in the environmental domain, conducted an informal review of imagery in publicly available persuasive climate messages. This involved examination of roughly 150 messages drawn from multiple campaigns, organizations, and websites. Images were collected from Google Images and other online databases (e.g., www.adsoftheworld.com and www.pinterest.com). Search terms were combinations of keywords including *climate change, global warming, sustainability, pollution, awareness, campaigns, advertising*, and *poster*. Search for any combination of keywords was stopped when a point of saturation was reached, meaning that no new campaign images were identified. The resulting set of images served as input for discussion among members of the research team.

2. Ratcliff (2021) urges reactance researchers to be as transparent as possible regarding their methodological choices (p. 1052). Hence, we elaborate our reasons for eliminating one item from the Dillard and Shen (2005) scale. We believe that when two conditions hold, a scale may be reduced in length without appreciable loss of validity. The first requires evidence that the items are substitutable methods of assessing a single latent construct. Such evidence is generally gleaned from some form of factor analysis. If that analysis demonstrates the unidimensionality of the item set, and the factor loadings do not differ by more that would be expected by sampling error, then any item or items (from the existing scale) can be eliminated to produce a short form. The second criteria, and the countervailing constraint, is reliability. One does not want to eliminate so many items that the item set becomes unreliable. But, the expected reliability of a reduced item set can be estimated from prior data using the Spearman-Brown prophecy formula. For example, if we take the mean alpha (.93) for the four-item threat scale reported in Dillard and Shen (2005), the prophecy formula predicts that a three-item scale will produce an alpha of .91. Thus, in the planning stage of the current study, we expected only a small decrement in reliability. The data produced a higher-than-expected value of .96.

3. In addition to the mean comparisons presented here, Supplemental Table S1 summarizes the means and standard deviations for all dependent variables by condition.

4. We reversed the sign on Rains’ (2013) coefficient so that it matches the scaling of our perceived effectiveness variable.

5. A different perspective—suggested by one of our reviewers—is to consider mean values. Looking at the anger component of reactance, the mean scores in both studies were below the mid-scale point. While low anger scores are typical in reactance research (e.g., see Acheme et al., 2024; Clayton et al., 2020; LaVoie et al., 2017; Quick & Stephenson, 2008; Tian & Solomon, 2023), they raise a question about the conceptualization of reactance (We thank Reviewer 2 for their comments on this topic). Previous research and the present paper have conceptualized reactance as a relative phenomenon, viewing it as a continuous variable that can range from zero to some higher value and that indicates the *degree* of reactance experienced by a participant. An alternative view is that experiencing *some* anger and *some* critical thoughts does not automatically qualify as reactance (e.g., just as feeling a bit uncomfortable when spotting a spider does not automatically qualify as arachnophobia). This perspective favors an absolute (or clinical) approach to reactance, where individuals are only considered reactant if they surpass some specific threshold. We are not aware of any reactance research that has developed this threshold idea. It may be worth considering whether it is meaningful to embrace the idea or a reactance threshold and, if so, what numerical value it should take. Additionally, clarification may be needed on what to call the psychological state when individuals experience some anger and critical cognitions, but remain below the threshold. As the relative and absolute approaches have their merits but are based on different conceptual foundations, we encourage future thinking that specifies the problem and the solution(s) more precisely.
