# Supplementary material for: When Visual Communication Backfires: Reactance to Three Aspects of Imagery
Source: Communic Res. 2025 Jan 9;52(5):683–713. doi: 10.1177/00936502241306707 (PMC12094521; doi:10.1177/00936502241306707)
Supplement: sj-docx-1-crx-10.1177_00936502241306707 – Supplemental material for When Visual Communication Backfires: Reactance to Three Aspects of Imagery [file sj-docx-1-crx-10.1177_00936502241306707.docx]

**Supplementary Tables and Figures**

**Table S-1**

*Study 1: Means and Standard Deviations of Study Variables by Condition*

| Image  Condition | Threat to  Freedom^a^ M (SD) | Reactance^b^ M(SD) | Perceived  Effectiveness^c^ M(SD) |
| --- | --- | --- | --- |
| Eye-Level | 2.362 (1.309) | .086 (1.473) | 2.828 (1.677) |
| Low Angle | 2.612 (1.317) | .719 (1.689) | 2.765 (1.686) |
| Thesis | 1.806 (1.078) | -.894 (1.022) | 4.960 (1.958) |
| Antithesis | 2.576 (1.340) | -.037 (1.443) | 4.439 (1.876) |
| Happy Expression | 2.069 (1.210) | -.559 (1.291) | 3.737 (1.811) |
| Angry Expression | 2.622 (1.413) | .685 (1.677) | 2.617 (1.592) |
| All Conditions | 2.341 (1.317) | .000 (1.565) | 3.558 (1.982) |

^a^Scale range = 1 to 5.

^b^z-scores.

^c^Scale range = 1-7.

*Note.* Because all of the data derive from a single sample with repeated measures, the statistics are for descriptive purposes only.

**Table S-2**

*Study 1: Correlations by Image Condition*

|  | PO | Condition | Threat | Reactance |
| --- | --- | --- | --- | --- |
| Camera angle (low angle = 1, eye level = 0) | | | | |
| Angle | .000 |  |  |  |
| Threat | .311 | .095 |  |  |
| Reactance | .164 | .196 | .494 |  |
| PE | -.305 | -.019 | -.260 | -.365 |
| (Anti)thesis (antithesis = 1, thesis = 0) | | | | |
| (Anti)thesis | .000 |  |  |  |
| Threat | .262 | .303 |  |  |
| Reactance | .247 | .325 | .531 |  |
| PE | -.357 | -.135 | -.342 | -.564 |
| Emotion Expression (angry = 1, happy = 0) | | | | |
| Emotion | .000 |  |  |  |
| Threat | .339 | .206 |  |  |
| Reactance | .190 | .385 | .508 |  |
| PE | -.256 | -.313 | -.300 | -.456 |

*Note.* PO = Political Orientation (1 = conservative, 0 = liberal); PE = Perceived Effectiveness. Because all of the data derive from a single sample, the statistics are presented for descriptive purposes only.

**Table S-3**

*Study 1: Threat to Freedom by Image Condition Based on Between-Persons Data*

| Image  Condition (*N*) | M (*SD*) | Mean  Difference^a^ | *p* value | *d* |
| --- | --- | --- | --- | --- |
| Eye-Level (42) | 2.817 (1.422) | -.204 | .501 | .15 |
| Low Angle (37) | 2.612 (1.253) |  |  |  |
| Thesis (32) | 1.823 (1.051) | .956 | .0005 | .69 |
| Antithesis (47) | 2.730 (1.524) |  |  |  |
| Happy (33) | 2.111 (1.085) | .907 | .004 | .82 |
| Angry (49) | 3.068 (1.234) |  |  |  |

^a^Differences were computed such that the low-predicted-threat cells (eye level, thesis, happy expression) were subtracted from the high-predicted-threat cells. Thus, positive values indicate higher levels of perceived threat for the cells that were predicted to produce higher threat levels.

**Table S-4**

*Study 2: Message Manipulation Examples and Predictor Coding*

| **Image Variation – Level** | **Message Level Variable Coding** | | | | |
| --- | --- | --- | --- | --- | --- |
|  | **High Threat Image** | **Antithesis Variation** | **Emotion Variation** | **Antithesis Present** | **Emotion Anger** |
| Camera Angle – Low-Angle | 1 | 0 | 0 | 0 | 0 |
| Camera Angle – Eye-Level | 0 | 0 | 0 | 0 | 0 |
| Antithesis –  Present | 1 | 1 | 0 | 1 | 0 |
| Antithesis – Absent | 0 | 1 | 0 | 0 | 0 |
| Emotion Expression – Anger | 1 | 0 | 1 | 0 | 1 |
| Emotion Expression – Happy | 0 | 0 | 1 | 0 | 0 |

**Figure S-1**

*Study 1: Histograms of Key Variables*

*
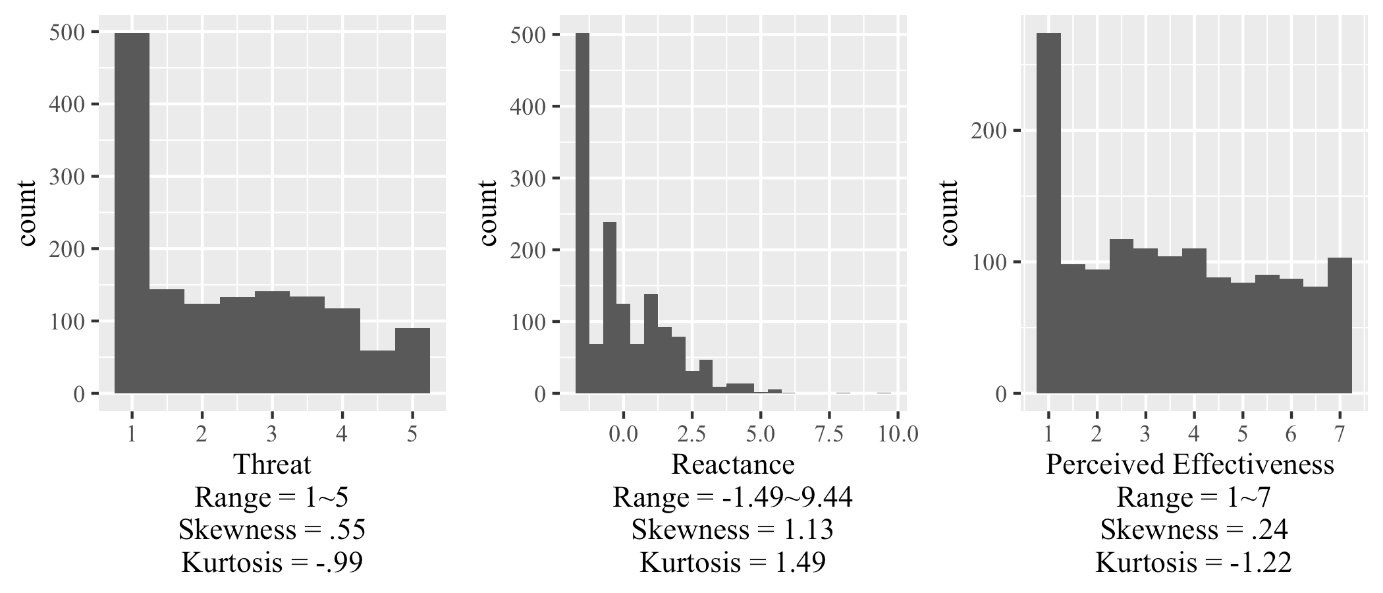
Note*. Data from all conditions were pooled to create these histograms (i.e., N = 240 X 6 image conditions = 1440 observations). The distributions for threat to freedom and perceived effectiveness are shown in the metrics in which they were measured (i.e., 1-5 and 1-7 respectively). For reactance, the data are the sum of z-scores for anger and critical cognitions.

**Figure S-2**

*Study 2: Low Angle Stimuli (Left) vs. Eye Level Stimuli (Right)*

| 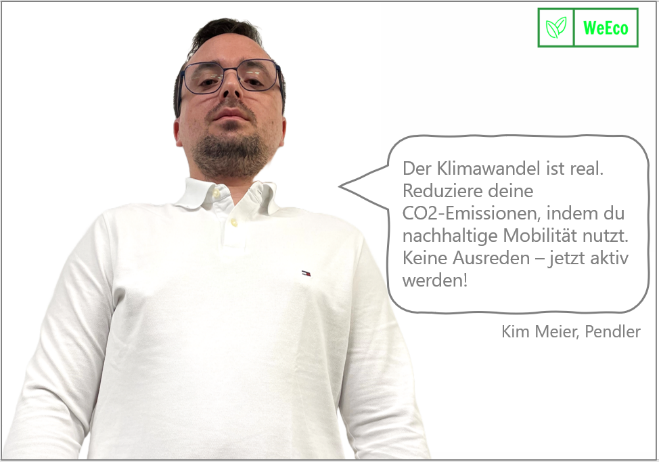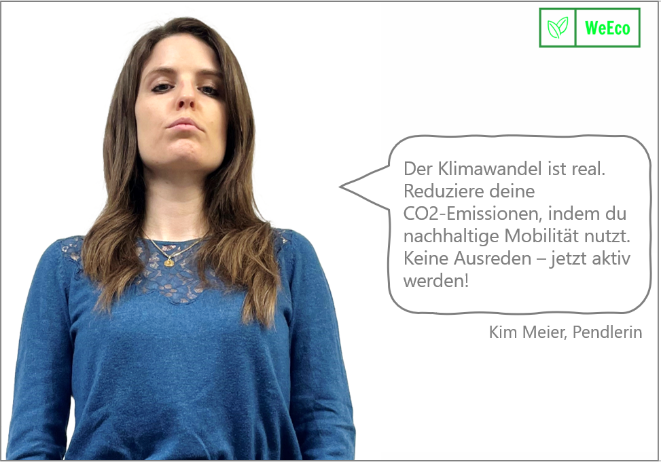Low camera angle | Eye level angle  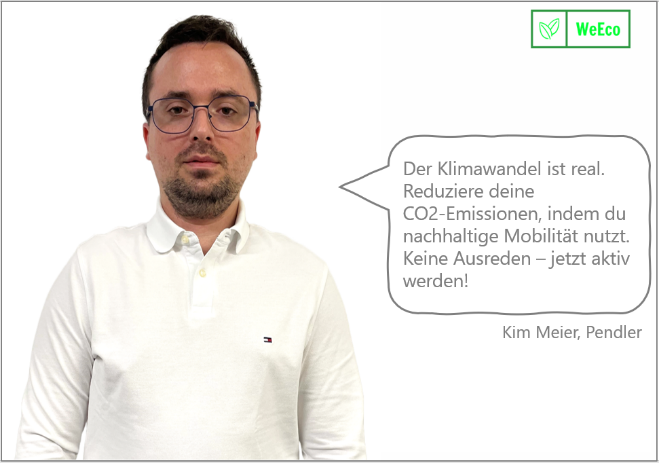  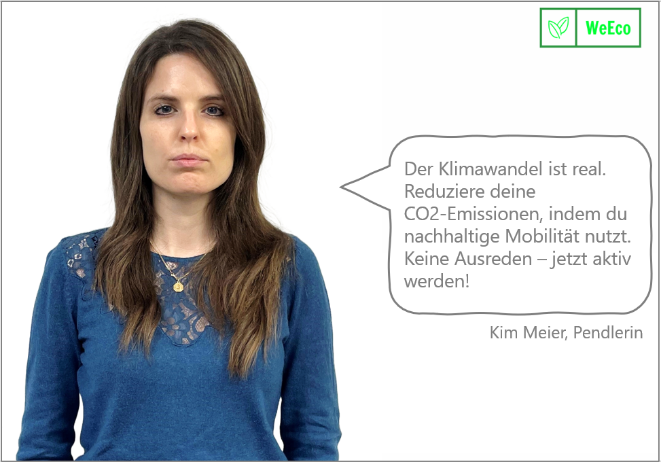 |
| --- | --- |

*Note*. English translation of the ad copy: “Climate change is real. Reduce your CO2 emissions by using sustainable mobility. No excuses – act now!”

**Figure S-3**


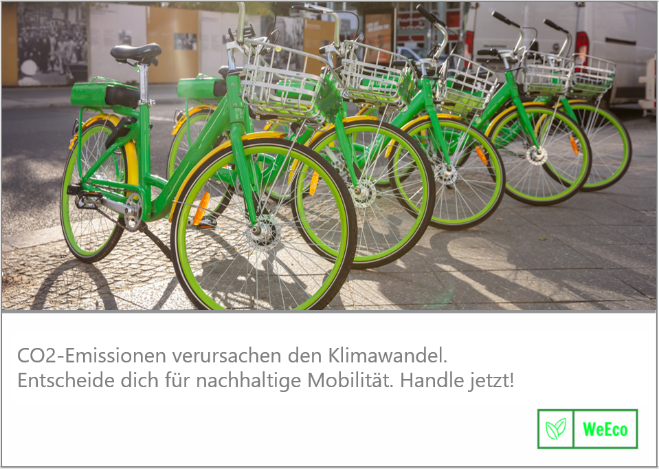
 *Study 2: Visual Antitheses (Left) vs. Theses (Right)*

| Antithesis | Thesis | |
| --- | --- | --- |
| 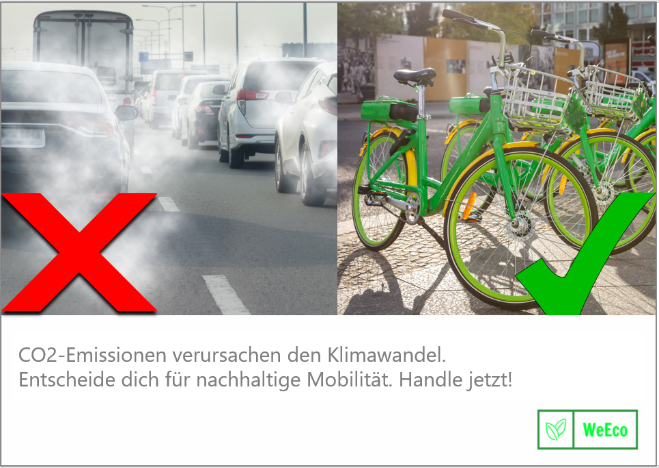  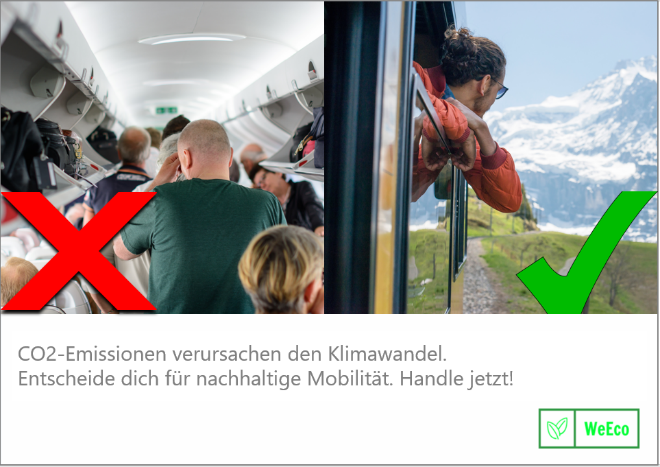 | | 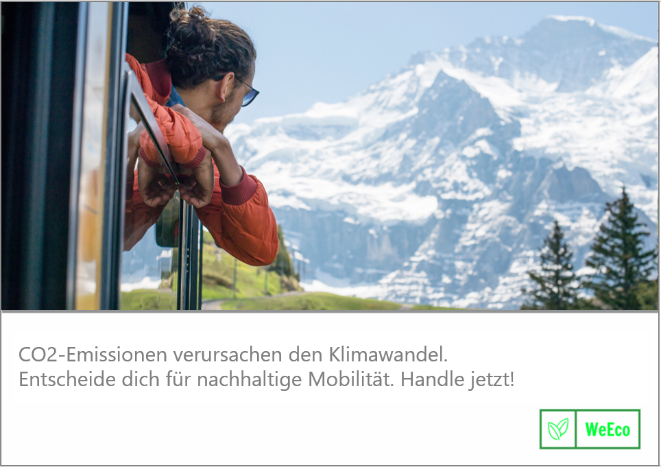 |

*Note*. Translation of the ad copy: “CO2 emissions cause climate change. Opt for sustainable mobility. Act now!”

**Figure S-4**

*Study 2: Angry-looking People (Right) vs. Happy-looking People (Left)*

| Angry facial expression | Happy facial expression |
| --- | --- |
| 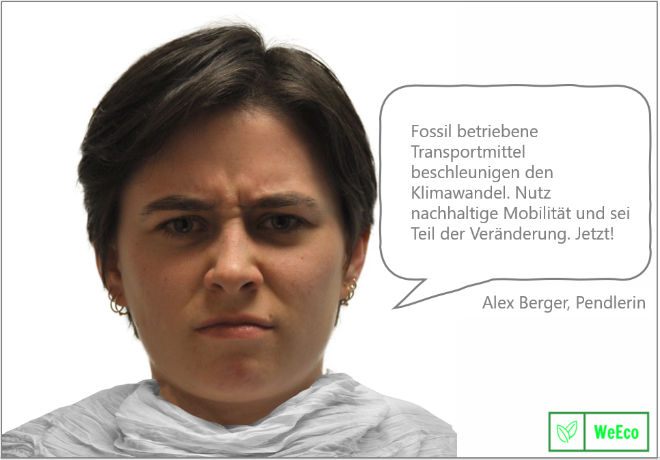  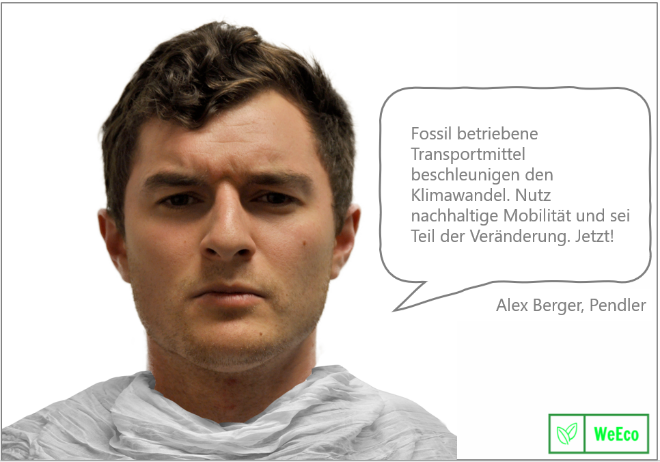 | 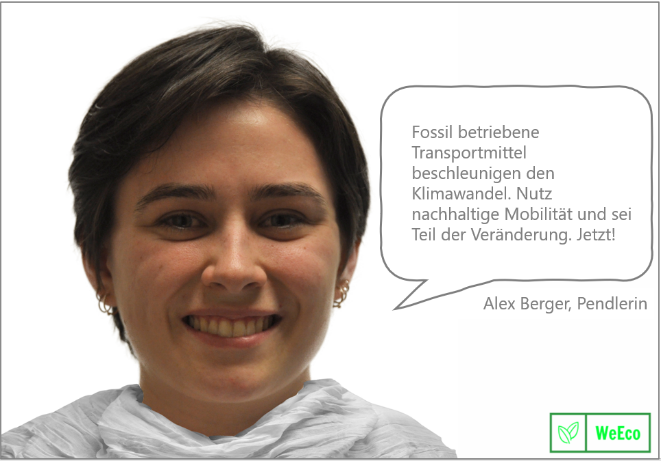  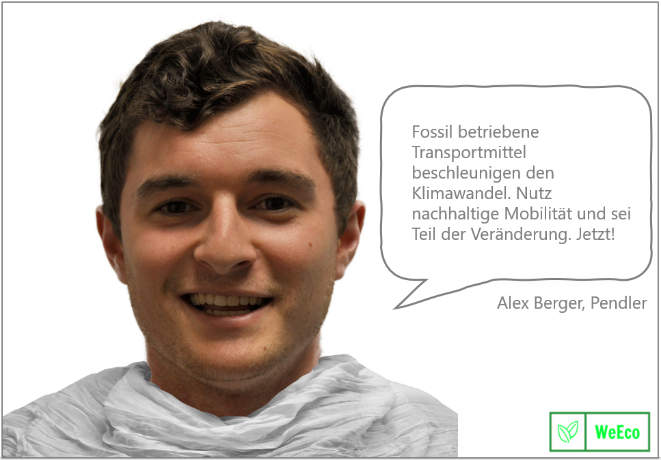 |

*Note*. English translation of the ad copy: “Fossil-fueled transportation accelerates climate change. Use sustainable mobility and be part of the change. Now!”

**Supplemental Appendix**

**Study 1: Alternative Models**

Although Model 4a showed good fit to the data, MacCallum et al. (1993) note the potential for other models fit the data equally well. It is advisable to test and, one hopes, eliminate rival interpretations of the data. We tested two alternative models, each of which varied the ordering of variables in the two-step reactance process. To strengthen the comparison, we advantaged both alternative models by removing all equality constraints from them.

The first alternative swapped the position of threat and reactance such that reactance 🡪 threat (cf., Figure 3). Fit statistics for this model were uniformly poor: χ2(108) = 432.643, p < .001, CFI = .852, TLI = .765, RMSEA = .112 [.101, .123], pCLOSE < .001, SRMR = .178. BIC = 13833.419.

In the second alternative model, the causal location of reactance and PE were exchanged such that PE 🡪 reactance (cf., Figure 3). This analysis yielded: χ2(108) = 312.455, p < .001, CFI = .907, TLI = .852, RMSEA = .089 [.077, .100], pCLOSE < .001, SRMR = .159. BIC = 13710.812. Although the RMSEA might be minimally acceptable under some circumstances, the other indices favored rejection of this alternative. We concluded that the causal ordering of variable in Model 4a was superior to both of the rival models, then proceeded to evaluate the results with respect to individual hypotheses.

# Between- and Within-person Designs in Studies of Reactance and Persuasion

Confidence in scientific conclusions grows when the effect of interest is observed across of range of settings, researchers, and methods. But, examination of the reactance literature reveals a nearly universal preference for between-person designs. Despite its popularity and utility, the post-test only design suffers from relatively low statistical power and from threats to internal validity that it cannot detect. The problem posed by statistical power arises, in part, because measurement at only one point in time cannot differentiate random variance from person-specific variance: The two sources of error are confounded. This leads to another problem, one that is not yet widely appreciated in communication science. This is the threat to internal validity, known as *causal heterogeneity*. It is the possibility that different causal processes are at work in different groups of subjects (Bullock et al., 2010).

To see this issue clearly it is helpful to reflect on what is wanted from an experiment versus what an experiment actually delivers. Imagine a 1 X 2 study intended to examine the effect of aggressive language on perceived threat to freedom. One message expresses the advocacy using forceful language, whereas the other appeal emphasizes the autonomy of the message recipient. The researchers seek to test the idea that the messages differ in their impact on perceived threat. To do that, they want the two groups to be equivalent on every possible dimension. If they are not, then critics can point to a difference and argue that the apparent effect of the messages is not due to the messages at all, but to that group difference. Thus, non-equivalent groups create alternative explanations that researchers want to eliminate. The best way to avoid the problem posed by non-equivalent groups would be to use the same participants in both cells at the same time (Pirlott & MacKinnon, 2016). The two groups would be completely equivalent. Regrettably, there are practical difficulties that preclude implementation of this approach. As so often happens in other walks of life, researchers must settle for less than what they desire (Jagger & Richards, 1969).

The conventional solution has been to put half of the participants in one cell and half in the other, then utilize the power of counterfactual logic. Movement from data patterns to substantive conclusions requires faith that the no-message control participants *would have* behaved the same as participants in the message-treatment condition *if* they had been assigned to that condition, and vice versa. Given that individuals are assigned to only one group (i.e., control *or* treatment), how participants might have performed in the other condition is not and cannot be known from this design. Rather, it must be assumed.

Whether or not that assumption is warranted depends on how strongly one believes that equivalent groups can be created prior to message exposure. Enter randomization — a procedure for assigning participants to groups such that each participant has an equal probability of being placed in either group. It has been reported that Fisher (1935) said that randomization “relieves the experimenter from the anxiety of considering innumerable causes by which the data may be disturbed” (Saint Mont, 2015, p. 1). He was surely right that it works to down-regulate negative affect in researchers. Yet, apart its therapeutic function, there is a clear difference between randomization as a process and randomization as an outcome (Owaru et al., 2022). The process deals in expected values, but outcomes often depart from expectations. The fact that the probability of assignment to either group is equal before assignment does not guarantee any particular distribution after assignment. And just as sampling theory would predict, the process of randomization can produce distributions that differ between cells, not only on the variables of interest, but also on all of the variables that go unmeasured (Krause & Howard, 2003; Saint Mont, 2015). Indeed, main effect differences are the simple case. Even if randomization produces identical distributions in the two cells for all the variables under study “the absence of confounding by main effects does not entail the absence of confounding by some interaction effects . . .” (Krause & Howard, 2003, p. 756). Collectively, these threats to internal validity go by the label *causal heterogeneity* (Bullock et al., 2010). One causal process takes place in one group and a different causal process in another. The post-test only design cannot assess causal heterogeneity because whatever differences might exist between groups are completely confounded with the treatment (or absence thereof). So, users depend on the assumption that randomization guarantees equivalent groups. But, in practice, it does not. All between-group reactance studies are open to this critique — which is to say, virtually all previous reactance studies.

As suggested earlier, one way to create equivalent groups would be to assign each participant to every cell in the design at the same time. Of course, we could not satisfy the “at the same time” aspect of this approach. We could, however, proceed with the knowledge that each group in Study 1 was perfectly matched with every other group. And, in associational terms (i.e., the path coefficients), the data revealed that the basic reactance process was directionally the same in every condition.^[[1]](#footnote-2)^ These results are evidence of causal homogeneity, evidence that we believe is unique to the study of persuasion and reactance.

One loose thread remains. Because we could not meet the “at the same time” criterion, there is the risk that our participants were not equivalent over time, that they changed in some way over the course of the experiment. Here we can rule out certain classes of temporal changes, such as developmental processes, as implausible given the (brief) duration of the experiment. We cannot, however, eliminate the possibility of short-term carryover effects. Still, the standard argument for alleviating concern about carryover effects is that the design was completely counterbalanced. Our layered randomization of participants to conditions should have been effective at reducing systematic bias. If randomization relieves anxiety about between-person designs, should it not do the same for within-person designs?

It is also possible to examine the results for evidence of carryover effects. We approached this question in four ways. One was to use the sequence of message exposures as a clustering variable in the computation of an intraclass correlation in Study 1 (there are 48 possible sequences).^[[2]](#footnote-3)^ This produced values that ranged between .032 and .104, an indication that small carryover effects were present in the data.

We also attempted, in Study 1, to fit linear and quadratic growth curve models for threat over time, reactance over time, and perceived effectiveness over time. None of these models fit. These results indicated no discernable trends across the six measurements. In other words, no evidence of carryover effects.

Moreover, we computed two-tailed *t*-tests of temporally adjacent means for threat, reactance, and perceived effectiveness in Study 1. For example, threat at t1 versus t2. Of the 15 possible comparisons, four showed significant differences (*p* < .05). One occurred for threat (a decrease between t1 and t2); one for reactance (a decrease between t1 and t2); and two for perceived effectiveness (decreases between t3 and t4 and between t5 and t6). So, while 11/15 cells showed no significant differences, 4/15 showed change in the same direction. Although we would be hard pressed to explain the results for perceived effectiveness, threat and reactance both showed increases in the same time period (from t1 to t2). Participants were less reactive to the second message than to the first. This could be a real difference. There was no indication that this carryover extended beyond t2.

Fourth, we included a viewing sequence variable to examine and control for order effects on the reactance process in Study 2 (first image = 1, last image = 6). Image position exerted an effect on threat, reactance, and perceived effectiveness, such that images viewed later in the experiment were rated as less threatening (β = -.109, p < .001) but induced more reactance (β = .073, p = .006), and they were viewed as more effective (β = .180, p < .001) than images positioned earlier in the viewing sequence.

Overall then, three of our four approaches showed some evidence of (small) carryover effects. Because they were counterbalanced across conditions, however, there is no reason to believe that they imperil the substantive conclusions of the project. Rather, the consistent pattern of findings across conditions, generated by the same participants, argues against the possibility of causal heterogeneity in the reactance process. We know of no other investigation in the persuasion/reactance literature that can make this claim.

We suspect that some readers will dismiss the threat of causal heterogeneity, perhaps because it is unfamiliar. Instead, they will focus the devil they know: The possibility of a demand effect. This concern began with Orne (1962), who warned that “. . . college students share (with the experimenter) the hope and expectation that the study in which they are participating will in some material way contribute to science and perhaps ultimately to human welfare in general” (p. 778). Due to their high-minded motives, researchers should expect that “. . . as far as the subject is able, he (sic) will behave in an experimental context in a manner designed to play the role of a "good subject" or, in other words, *to validate the experimental hypothesis* (italics in the original) (p. 778). Thus, demand effects are a source of systematic bias that offer a theoretically barren alternative explanation for any given set of results.

In broader terms, Orne’s (1962) thinking assumes that two conditions must be present for demand effects, one cognitive and one motivational. First, participants must be able to infer the research hypothesis. In the context of our Study 1, they could surely deduce that we were interested in how their responses to different image types might vary. But, the idea that they could predict the direction of the individual hypotheses seems rather far-fetched. If participants do seek to aid the experimenter, yet lack information regarding the direction of the research hypothesis, the result is random error, not a demand effect.

The second necessary condition centers on motivation. Orne’s (1962) quaint view of participants’ goals has all the marks of a benevolent, but self-absorbed psychologist engaged in projection, a view that might be summarized as “I have the goal of aiding humanity, so they must have the goal of aiding me in that endeavour.” Other writers take a different view. Cook et al. (1970) raise the possibility of participants whose goal is to provide data that is contrary to the hypothesis. In contrast, Frank (1998) contends that researchers are full of themselves: Participants simply do not care about what they are trying to achieve. We suggest that there is merit to all of these positions in roughly equal measure. In research studies and in life, some people will be in favor, some against, and a large middle will be apathetic. The effect is nonsystematic error, not a demand effect.

Are there any data that might speak to this claim? A systematic review of the literature on demand effects found only seven empirical studies (McCambridge et al., 2012), all but one of which used non-experimental designs. The authors bemoan both the quality and quantity of relevant data before concluding that their investigation “. . . calls into question whether the demand characteristics construct is useful for wider research purposes” (e39116), that is, non-laboratory studies.

Addressing this question, Mummolo and Peterson (2019) set themselves to the task of empirically examining the potential for demand effects in online surveys. Five surveys on different topics were conducted that involved over 12,000 respondents. Control conditions that attempted to obscure the hypothesis were compared to conditions in which participants were provided with an explicit hypothesis (e.g., “We expect that people will be more likely to choose an article if the news source offering it is known to favor their preferred political party”) (p. 522). The researchers found no evidence of demand effects, even among Amazon Turk workers whose output can be evaluated by the researcher. This study also highlights a potentially important scope condition. Orne’s (1962) concerns derive wholly from laboratory interactions between experimenters and college undergraduates. Even if we assume that participants do want to please researchers, whom do they have in mind when taking part in an online experiment?

Data and reason converge on the conclusion that there is no demand-effects monster hiding under the bed. Critics might say that Yes, that might be generally true, but how can we be sure that it is not the case in *this* research? For such persons, nothing other than data from a between-persons study will suffice. To address their concerns, we conducted a between-subjects analysis in Study 1 and added a between-subjects component to the randomization process in Study 2. We turn to Study 1 first. Because participants were randomized to image type, then to image variation, everyone in the sample viewed one of the six image variations first, that is, prior to viewing the other five variations. When the later exposures are removed the result is a 1 X 2 between-persons experiment for each of the three image contrasts. There are far fewer degrees of freedom because the 240 participants are (randomly) spread over the six cells. Given an average cell size of (240/6 =) 40, power to detect a difference at α = .05, two-tailed, was .16, .69, and .97 for small, medium, and large effects respectively (i.e., *d* values of .20, .50, and .80). As Table S-3 shows, the findings for the between-persons analyses replicate those of the within-person analyses for (anti)thesis and emotional expression. No indication of a monster under the bed.

For camera angle, however, the between-persons result was nonsignificant while the same comparison in the within-persons data was significant: The mean differences ran in opposite directions. Here the two designs seem to give conflicting answers. One obvious explanation for the discrepancy is that the small effect size observed in the within-persons analysis (equivalent to *d =* .19) was not detected in the between-persons analysis due to the diminished power in the latter. Indeed, if we assume that .19 is the true effect size, then power with 80 participants was a dismal .13. To achieve power of .80, total *N* would have to be increased to 872. From the standpoint of drawing substantive inferences, it seems safe to say that if there is a genuine effect for camera angle, it is very small. On this point, the results from the within and between analyses are in perfect agreement. As to which of the two results is more accurate, we would put our money on within-persons given the smaller standard errors and the absence of any compelling reason to believe that those findings are threatened by demand effects. After all, it does not seem plausible that demand effects were present for camera angle, but not for (anti)thesis and emotional expression.

In Study 2, we added a between-subjects element to the randomization process. Participants were exposed to the three visual features in random order [i.e., camera angle, (anti)thesis, emotion]. Within each visual feature, they were randomly assigned to *either* the high threat condition [i.e., low shot, antithesis present, angry face] *or* the low threat condition [i.e., eye level shot, antithesis absent, happy face]. Subsequently, participants were presented with two stimuli within the assigned condition, displayed in random order. Because no one saw both threat levels within the same features, the effects are between-subjects. The results consistently support the veracity of the within-subjects findings from Study 1, demonstrating the reactance-inducing potential of all image variations under scrutiny. Taken together, we do not wish to leave the impression that between-person designs are not valuable. They are, and will continue to be, important tools for the generation of knowledge. But they are not the gold standard. Every design has strengths and weaknesses. Strong scientific conclusions depend on the use of a diversity of methods that balance threats to validity over the long run.

**References**

Bullock, J. G., Green, D. P., & Ha, S. E. (2010). Yes, but what’s the mechanism? (Don’t

expect an easy answer). *Journal of Personality and Social Psychology, 98*(4)*,* 550-558. https://doi.org/10.1037/a0018933

Cook, T. D., Bean, J. R., Calder, B. J., Frey, R., Krovetz, M. L., & Resiman. S. R. (1970). Demand characteristics and three conceptions of the frequently deceived subject. *Journal of Personality and Social Psychology*, *14*(3), 185–194. https://doi.org/10.1037/h0028849

Fisher R. A. (1935). *The design of experiments* (8th edition). New York: Hafner.

Frank, B. L. 1998. Good news for the experimenters: Subjects do not care about your welfare. *Economics Letters, 61*(2)*,* 171–174. https://doi.org/10.1016/S0165-1765(98)00162-1

Jagger, M., & Richards, K. (1969). You can’t always get what you want. *Let It Bleed.* London: Decca.

Krause, M. S., & Howard, K. I. (2003). What random assignment does and does not do.

*Journal of Clinical Psychology, 59*(7)*,* 751-766. https://doi.org/10.1002/jclp.10170

MacCallum, R. C., Wegener, D. T., Uchino, B. N., & Fabrigar, L. R. (1993). The problem of equivalent models in applications of covariance structure analysis. *Psychological Bulletin, 114*(1), 185–199. https://doi.org/10.1037/0033-2909.114.1.185

McCambridge J., de Bruin M., & Witton J. (2012). The effects of demand characteristics on research participant behaviours in non-laboratory settings: A systematic review. *PLoS ONE, 7*(6), e39116. https://doi.org/10.1371/journal.pone.0039116

Mummolo, J., & Peterson, E. (2019). Demand effects in survey experiments: An empirical assessment. *American Political Science Review, 113*(2)*,* 517-529. https://doi.org/10.1017/S0003055418000837

Orne, M. T. (1962). On the social psychology of the psychological experiment: With particular reference to demand characteristics and their implications. *American Psychologist, 17*(11)*,* 776–783. https://doi.org/10.1037/h0043424

Owora, A. H., Dawson, J., Gadbury, G., Mestre, L. M., Pavela, P., Mehta, T., Vorland, C. J., Xun, P., & Allison, D. B. (2022). Randomization can do many things – but it cannot “fail.” *Significance, 19*(1)*,* 20-23. https://doi.org/10.1111/1740-9713.01609

Pirlott, A. G., & MacKinnon, D. P. (2016). Design approaches to experimental mediation. *Journal of Experimental Psychology, 66,* 29-38. https://doi.org/10.1016/j.jesp.2015.09.012

Saint Mont, U. (2015). Randomization does not help much, comparability does. *PLoS ONE,* *10*(7)*,* e0132102. https://doi.org/10.1371/journal.pone.0132102

1. This point does not apply equally to all paths, but it is complicated by the fact that some paths were rejected by our analysis of alternative models. A more complete expression of the argument and its limits can be found in Pirlott and MacKinnon (2016) and related literature. [↑](#footnote-ref-2)
2. Our two-layered randomization process involved six possible orders at the between-image variation level (i.e., arranging (anti)thesis, emotion, and camera angle), and two possible orders at each within-variation level for all three variations (e.g., happy followed by angry expression or reverse). This results in 6 × 2^3^ = 48 possible orders. [↑](#footnote-ref-3)
